# Stimulation of Germination of Freshly Collected and Cold-Stored Seeds of *Ambrosia artemisiifolia* L.

**DOI:** 10.3390/plants11141888

**Published:** 2022-07-20

**Authors:** Maja Šćepanović, Laura Košćak, Laura Pismarović, Valentina Šoštarčić

**Affiliations:** 1 Department of Weed Science, Faculty of Agriculture, University of Zagreb, Svetošimunska 25, 10000 Zagreb, Croatia; mscepanovic@agr.hr (M.Š.); vsostarcic@agr.hr (V.Š.); 2 Department of Agriculture and Nutrition, Institute of Agriculture and Tourism, Karla Huguesa 8, 52440 Poreč, Croatia; laura@iptpo.hr

**Keywords:** common ragweed, ethephon, GA_3_, growth abnormalities, seed conditioning, thiourea

## Abstract

Herbicides are the most commonly used means of controlling the growth of *Ambrosia artemisiifolia* L. Their constant use has led to the development of resistant populations. They can be evaluated by studying seed germination and the corresponding grown plants, but *A. artemisiifolia* exhibits seed dormancy, preventing germination and delaying research. Here, we developed a simple and rapid method to stimulate germination of freshly collected or stored *A. artemisiifolia* seeds. The germination of *A. artemisiifolia* freshly collected/stored seeds was evaluated after storage, stratification, and chemical treatments (ethephon, gibberellic acid (GA_3_), thiourea, KNO_3_). Ethephon or ethephon + GA_3_ improved freshly collected seed germination by 88 and 95%, respectively, and germination of stored seeds by 78 and 80%, respectively. In addition, placing the seeds of *A. artemisiifolia* in ethephon, GA_3_, ethephon + GA_3_, or thiourea solutions caused the freshly collected seeds to germinate faster than stored seeds or nontreated seeds. In contrast, the conditioning of seeds in these solutions favored germination of stored seeds, especially when ethephon + GA_3_ or GA_3_ was used. Imbibition of the freshly collected *A. artemisiifolia* seeds in a mixture of ethephon and GA_3_ can effectively overcome primary dormancy when rapid experimental results are needed. For seeds requiring prolonged storage, conditioning in ethephon, GA_3_, or thiourea solutions may be applied to promote germination.

## 1. Introduction

*Ambrosia artemisiifolia* L. (common ragweed) is a highly competitive summer annual weed that originated from North America and occurs in all arable crops that are sown or planted in mid-spring [1]. Its wide range of tolerated conditions and high germination rates across a broad spectrum of temperatures [2], photoperiods, pH, and soil salinity levels, as well as its high variability, must be considered crucial conditions for its rapid spread [3], which poses a serious threat to public health [4]. Extensive research in mainland Croatia has shown that *A. artemisiifolia* is the most common broadleaf weed among arable crops [5]. Herbicides are usually used to control its growth, but their constant use has led to resistant biotypes [6].

The efficacy of herbicides and the development of herbicide-resistant populations can be evaluated by studying germinated seeds and the corresponding grown plants. However, freshly collected *A. artemisiifolia* seeds exhibit primary dormancy, also known as endogenous (physiological) dormancy [7], which can inhibit germination for more than six months, thus prolonging the time that is required for plant growth in greenhouse experiments [8]. Given that seed dormancy release occurs naturally at low temperatures and in humid conditions during the winter, freshly collected seeds can be stratified at 4 °C in moist sand. However, this method is time-consuming, requiring up to 12 weeks to promote germination [9].

Stratified *A. artemisiifolia* seeds germinate when the balance changes between the growth-promoting hormone gibberellic acid (GA_3_) and the growth-inhibiting hormone abscisic acid [7,9,10,11]. Ethylene also promotes seed germination of many species by reducing the sensitivity of the seeds to endogenous abscisic acid [12]. Therefore, plant growth hormones or their combination have been used to shorten primary dormancy as well as to initiate germination and plant growth. In particular, ethephon (synthetic form of ethylene in liquid state), GA_3_, and kinetin (cytokinin) have been used to release primary and secondary dormancy in *A. artemisiifolia* seeds, but a single hormone application does not significantly stimulate germination. In contrast, the combination of ethylene and gibberellin significantly promotes the germination of some weed species seeds [13,14,15]. The germination of *A. trifida* seeds can also be significantly promoted using chemical agents instead of water, but seedlings with abnormal growth occasionally develop [16].

Other methods have been used to release the dormancy of various *Ambrosia* species, including nitrate fertilizers, mechanical scarification, and embryo extraction [9,16,17]. Among them, embryo excision from *A. trifida* seeds may be the most effective method, but it is laborious and, therefore, unsuitable for large numbers of seeds. To our knowledge, embryo excision has not yet been tested on *A. artemisiifolia* seeds.

Growth-promoting hormones have been used to break dormancy in *R. crispus* seeds [15]. However, the effects of dormancy-breaking growth-promoting hormones or chemicals on their germination and normal plant development have not yet been reported. Therefore, in this study we examined the response of freshly collected and stored *A. artemisiifolia* seeds to dormancy-breaking methods, with the aim of developing a simple and time-efficient method for stimulating the germination of *A. artemisiifolia* seeds. The effective stimulation of germination in dormant *A. artemisiifolia* seeds would help support future investigations into the effect of herbicides on *A. artemisiifolia* and consequently into overcoming herbicide resistance.

Freshly collected seeds exhibit primary dormancy [7], while prolonged seed storage can lead to seed senescence (loss of vigor) and plant death [18]. Laboratory experiments were first performed to investigate the effects of seed storage, cold stratification, chemical treatments, and their combination on the germination rate and germination dynamics of both seed types. The most effective dormancy-breaking methods that were identified in the laboratory experiments were then applied under greenhouse conditions to detect whether they give rise to growth abnormalities during the emergence and early growth of *A. artemisiifolia* seeds.

## 2. Results

### 2.1. Laboratory Experiments

The laboratory experiments indicated significant differences in the germination of freshly collected and cold-stored *A. artemisiifolia* seeds depending on the duration of cold storage, stratification time, or type of chemical treatments (Table 1). A significant difference was found for the adjusted means of the two factors that were studied, freshly collected/cold-stored seeds and treatments, and for the interaction between these factors.

#### 2.1.1. Seed Storage

**Figure 1:**
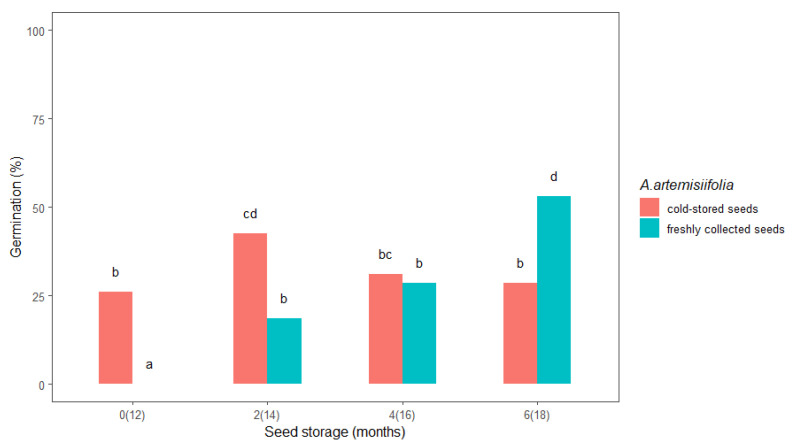
The effect of storage duration on freshly collected and cold-stored *Ambrosia artemisiifolia* seeds. The estimated marginal means of eight replicates are shown (standard error = 3.1). The means that are labelled with the same letter are not significantly different (*p* < 0.05) according to Tukey’s test. Numbers on the x-axis indicate the months of seed storage for the freshly collected seeds and the total months of storage in parentheses for the cold-stored seeds.

Prolonged cold storage seemed to favor the *A. artemisiifolia* seeds that were already in cold storage compared to the freshly collected seeds. A significant difference in the germination percentage was observed between freshly collected and cold-stored seeds in the first test (0, i.e., 12 months) and after two (14) and six (18) months of storage (Figure 1). The freshly collected seeds did not germinate in the first test, while the cold-stored seeds had a germination rate of 26%, and after two months of storage, the freshly collected and cold-stored seeds germinated at 18.5% and 42.5%, respectively. In general, the germination rate after zero, two, and four months was higher for the cold-stored seeds. Interestingly, germination of the freshly collected seeds increased from 0% after zero months of storage to 53% after six months, while germination of the cold-stored seeds decreased after four and six months. In addition, germination of the cold-stored seeds after six months of storage was almost half (28.5%) that of the freshly collected seeds after six months of storage.

#### 2.1.2. Cold Stratification

**Figure 2:**
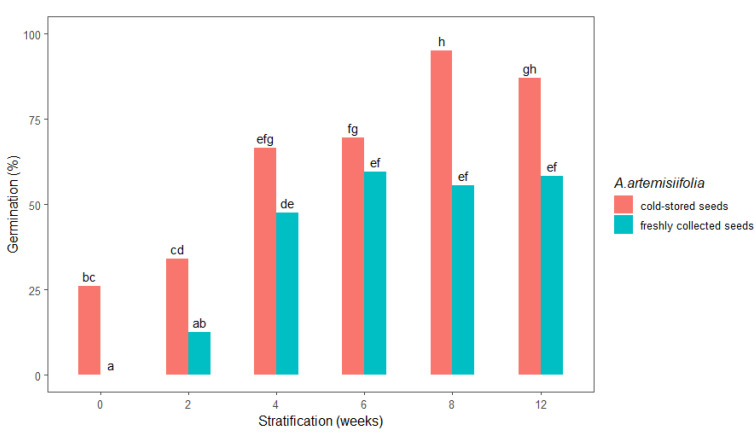
Total germination 14 days after sowing of freshly collected or cold-stored *Ambrosia artemisiifolia* seeds at different stratification intervals (0, 2, 4, 6, 8, and 12 weeks). The estimated marginal means of eight replicates are shown (standard error = 4.49). Means following the same letter are not significantly different (*p* < 0.05) according to Tukey’s test.

The germination of stratified *A. artemisiifolia* seeds was highly dependent on the duration of the stratification period and the studied freshly collected or cold-stored seeds (Figure 2). In particular, no germination occurred in the unstratified freshly collected seeds, whereas their germination was promoted as the stratification period increased. Stratification for shorter than four weeks did not satisfactorily break the seed dormancy (Figure 2), but germination of about 55% was achieved after 6, 8, and 12 weeks of stratification, respectively. In contrast, the germination percentage of unstratified cold-stored *A. artemisiifolia* seeds (26%) was significantly higher than that of the unstratified freshly collected seeds (0%). Increasing the duration of stratification promoted germination of the cold-stored seeds, with significantly higher germination rates after 8 and 12 weeks of stratification of 95% and 87% recorded, respectively. Statistical analysis further confirmed that there was no significant difference in the germination of the freshly collected seeds that were stratified for 4, 6, 8, or 12 weeks (Figure 2).

#### 2.1.3. Chemical Treatments

**Figure 3:**
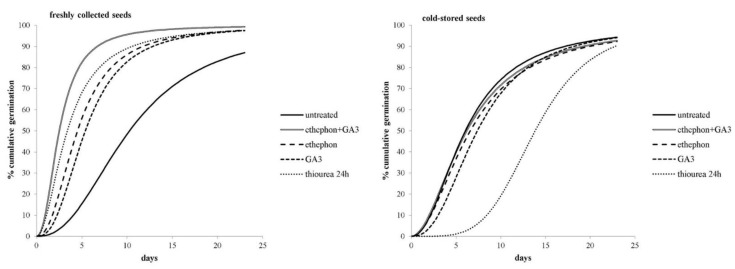
Cumulative germination over 14 days of the freshly collected or cold-stored seeds of *A. artemisiifolia* that were treated or not treated with chemical treatments. The lines represent logistic models that were generated with the statistical program Bioassay (97).

**Figure 4:**
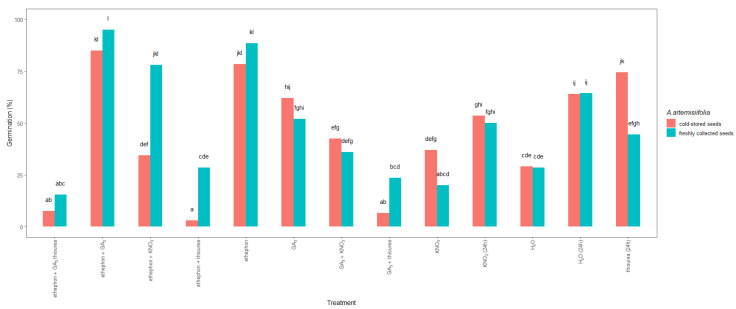
Total germination 14 days after sowing of freshly collected or cold-stored *Ambrosia artemisiifolia* seeds that were imbedded or treated with growth-promoting hormones or fertilizers. The estimated marginal means of eight replicates (standard error = 3.35) are shown. According to Tukey’s test, means following the same letter are not significantly different (*p* < 0.05). No germination was observed in seeds that were treated with thiourea + KNO_3_ or GA_3_ + thiourea + KNO_3_; data not shown.

Freshly collected or cold-stored *A.* *artemisiifolia* seeds were also treated with various growth-promoting chemicals, and the effects on germination were determined. Although cumulative seed germination was significantly affected by the type of chemical treatment that was used (Figure 3), it showed a stronger dependence on the time of seed collection and storage (Figure 4).

In general, the germination of nontreated freshly collected seeds started an average of two days earlier, and reached 50% at 4.2 days earlier, than the cold-stored seeds (Figure 3). Further treatment of freshly collected seeds with ethephon, GA_3_, ethephon + GA_3_, or thiourea significantly accelerated their germination compared to the nontreated seeds. For example, nontreated seeds reached 50% germination after 10.4 days, while the chemically-treated seeds required only 2.5–5.4 days (Figure 3). Conversely, the cold-stored seeds that were imbedded in thiourea took more than twice as long as the nontreated seeds to achieve 10% and 50% germination. Similar results were also found for GA_3_-treated cold-stored seeds, whereas the germination dynamic of cold-stored seeds that were treated with ethephon or ethephon + GA_3_ was similar to that of the untreated seeds.

Significant differences were also observed between the studied seeds (Figure 4). In particular, the germination of the freshly collected seeds that were treated with ethephon + KNO_3_ reached nearly a maximum, 78%, whereas a germination of only 34% was achieved in the similarly treated cold-stored seeds. In contrast, imbibition in thiourea resulted in 74% germination of the cold-stored seeds but only 44% germination of the freshly collected seeds. Moreover, when ethephon or ethephon + GA_3_ was used, the germination of the cold-stored and freshly collected *A.* *artemisiifolia* seeds improved to 78–95%, with the freshly collected seeds showing higher germination than the cold-stored seeds. In contrast, the application of nitrogen fertilizers or their combination with hormones resulted in significantly lower germination compared to other treatments, and a significant interaction was observed between the type of treatment and the seed collection time and storage (Table 1). Hormones and fertilizers were also used as a tank mix (ethephon + thiourea, ethephon + GA_3_ + thiourea, or GA_3_ + thiourea), but an antagonistic effect was observed, leading to low germination in both the freshly collected and cold-stored seeds.

**Figure 5:**
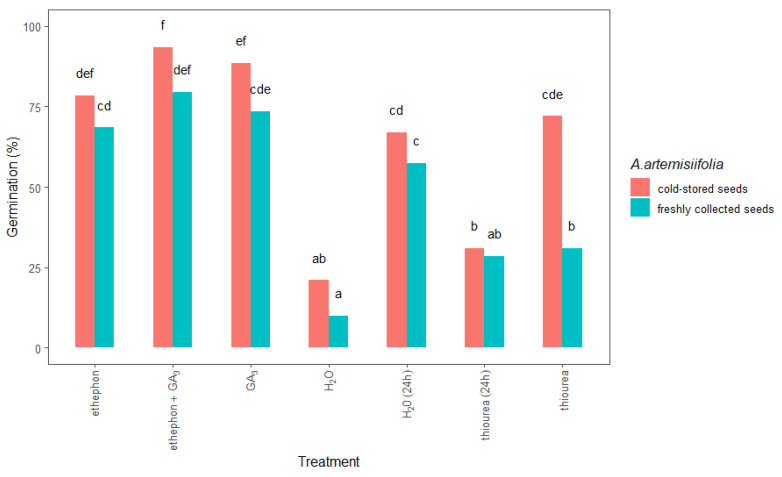
Total germination at 14 days after sowing freshly collected or cold-stored *Ambrosia artemisiifolia* seeds that were conditioned in water or chemical solution. The estimated marginal means of eight replicates (standard error = 3.35) are shown. According to Tukey’s test, the means following the same letter are not significantly different (*p* < 0.05). No germination was observed in seeds that were treated with thiourea + KNO_3_ or GA_3_ + thiourea + KNO_3_; data not shown.

Furthermore, conditioning in chemical solutions significantly improved the germination of the freshly collected and cold-stored *A. artemisiifolia* seeds compared to both types of nontreated seeds (Figure 5). In all cases, this process was more effective on the cold-stored than on the freshly collected seeds, especially when the seeds were conditioned with ethephon + GA_3_ (93% cold-stored vs. 79% freshly collected) or GA_3_ (88% vs. 73%). The application of ethephon alone also promoted the germination of cold-stored and freshly collected seeds by 78% and 68%, respectively. Interestingly, the germination of the cold-stored seeds was significantly higher (72%) than that of the freshly collected seeds (34%) after conditioning with thiourea.

### 2.2. Greenhouse Experiment

The laboratory experiments indicated that imbibition or conditioning in ethephon, GA_3_, ethephon + GA_3_, or thiourea were the most effective dormancy-breaking methods for *A. artemisiifolia* seeds. Therefore, these treatments were further applied in a greenhouse experiment to examine their effect on the emergence and development of *A. artemisiifolia* plants.

**Figure 6:**
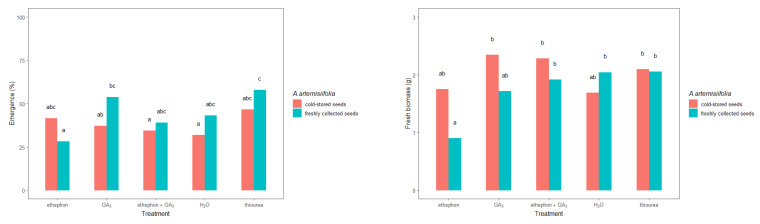
Initial growth parameters of young *Ambrosia artemisiifolia* plants that were grown from freshly collected or cold-stored seeds under various chemical treatments or conditioning in the greenhouse. The estimated marginal means of 16 replicates are shown for the freshly collected seeds (standard error = 4.19) and cold-stored seeds (standard error = 0.201). According to Tukey’s test, means following the same letter are not significantly different (*p* < 0.05).

The freshly collected and cold-stored seeds responded differently to the applied treatments, consistent with a treatment–seed interaction (Table 2). In general, the freshly collected *A. artemisiifolia* seeds emerged in greater numbers than the cold-stored seeds in most cases (Figure 6). However, a significant difference was observed only in the freshly collected seeds that were treated with ethephon + GA_3_ and thiourea. Moreover, only these two treatments resulted in emergence that was greater than 50%: 53.7% for ethephon + GA_3_ and 57.8% for thiourea.

The fresh biomass of *A. artemisiifolia* differed depending on the treatment or seed. In particular, freshly collected seeds that were treated with GA_3_ or conditioned in thiourea developed heavier seedlings (2.38 g and 2.52 g, respectively) than the nontreated seeds (1.89 g). Similar differences were also observed in the cold-stored seeds that were treated with ethephon (2.23 g), ethephon + GA_3_ (2.89 g) or GA_3_ (2.98), or conditioned in thiourea (2.63 g). In contrast, no significant differences were observed in the heights of plants that developed from the freshly collected and cold-stored seeds, except for freshly collected seeds that were conditioned in ethephon (5.07 cm) and cold-stored seeds that were conditioned in water (7.80 cm), which developed significantly lower plants than the respective nontreated seeds (9.03 and 8.60 cm, respectively). None of the other treatments led to significant differences in plant height.

**Figure 7:**
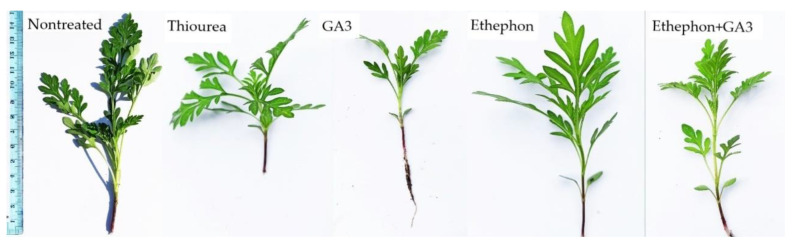
*Ambrosia artemisiifolia* young plants showing slight deformations upon chemical treatments.

Growth-promoting chemicals also led to abnormalities in the growth of *A. artemisiifolia*. For example, internodes formed between the seedling and the first pair of true leaves on seven plants that were grown from ethephon + GA_3_-treated cold-stored seeds. In addition, small deformations were observed on the first leaves of four plants that were grown from seeds that were imbedded or conditioned with GA_3_ or ethephon + GA_3_. In contrast, no deformations were detected in plants that derived from seeds that were imbedded or conditioned in thiourea (Figure 7).

## 3. Discussion

In this study, we investigated the ability of growth-promoting chemicals to break the dormancy of freshly collected or cold-stored *A. artemisiifolia* seeds. Although numerous studies have reported different germination patterns between different populations, probably due to their different dormancy levels [8] or genetic variability [19,20], the present study may be the first report on the susceptibility of *A. artemisiifolia* seeds to dormancy-breaking methods.

Our results indicate that the germination of *A. artemisiifolia* seeds depends significantly on the seed age. Specifically, only 26% of seeds that were stored at 4 °C for 12 months germinated (Figure 1), while further extension of the cold storage for six months did not improve their germination. This result is inconsistent with earlier studies, where the storage of *A. artemisiifolia* seeds at 4 or 5 °C for extended periods was found to break dormancy [21]. In contrast, 53% of the freshly collected seeds in our study germinated after six months of cold storage (Figure 1), implying that the seed age may limit germination due to the loss of viability [16,22,23]. Our findings were confirmed by the tetrazolium (2,3,5-tryphenyl tetrazolium chloride) test, where the cold-stored seeds showed lower viability than the freshly collected seeds (data not shown). Moreover, 50% and 90% germination occurred more than 4 and 8.6 days earlier, respectively, with the freshly collected seeds than with the cold-stored seeds (Figure 3), suggesting that germinating seeds should be obtained quite soon after harvest and that an efficient method should be developed to overcome dormancy in freshly collected seeds.

The stimulating effect of ethephon, GA_3_, thiourea, or KNO_3_ on the germination of *Ambrosia trifida* seeds has already been reported [17]. The present study extends the literature by showing that direct application or conditioning of *A. artemisiifolia* seeds with these chemicals has different effects on seed germination depending on the type of growth-promoting agents and the seed storage conditions. In particular, the application of ethephon or ethephon + GA_3_ either directly to the Petri dishes or as seed conditioning promoted the germination of *A. artemisiifolia* seeds more than the other treatments, and the effect was greater on the freshly collected seeds than on the cold-stored ones. Conversely, conditioning in a chemical solution (GA_3_ or thiourea) improved germination of the cold-stored seeds more than that of the freshly collected seeds (Figure 4), as already reported for *A. trifida* that were conditioned in water or chemical solution [16]. We also found that hormones (ethephon and GA_3_) were more effective than nitrogen fertilizers (KNO_3_ and thiourea) on the germination of freshly collected *A. artemisiifolia* seeds (Figure 4). Although nitrogen fertilizers can break the inhibition of seed germination, they cannot directly break dormancy, which makes them more effective at lower dormancy levels [24]. This difference between the chemical agents could explain the higher germination of the cold-stored seeds compared to the freshly collected seeds after imbibition in KNO_3_ or thiourea (Figure 4). In contrast, growth-promoting hormones had a better effect on the freshly collected *A. artemisiifolia* seeds, as the exogenous application of ethephon + GA_3_ or ethephon stimulated seed germination by 95% and 88%, respectively. These results are consistent with previous studies, where the imbibition or conditioning of cold-stored *A. trifida* seeds with GA_3_ did not improve germination compared to treatment with water [16]. Using higher GA_3_ concentrations is unlikely to help, since it may exacerbate prolonged internode growth [25]. As an alternative approach, GA_3_ has been used in a tank mixture with ethephon or kinetin, which moderately promoted germination [15]. In the present study, freshly collected or cold-stored *A. artemisiifolia* seeds were conditioned in a GA_3_ solution, which led to 88% and 73% germination, respectively, probably due to the leaching of these compounds during conditioning [16]. Although using sandpaper to release embryos from seeds requires time and skill, we found that it can significantly promote the germination of *A. artemisiifolia* seeds when GA_3_ is used for conditioning (Figure 5). However, adding other chemicals (ethephon + GA_3_ or thiourea) to the solution did not lead to greater germination than when seeds were exposed to the same chemicals by imbibition or exogenous application. Similar results have been reported for *A. trifida*, where seeds that were conditioned in a chemical solution developed almost four times fewer germinated seeds than seeds that were conditioned in water [16]. Based on these results, we conclude that the seed conditioning process is favorable only for cold-stored seeds that are treated with GA_3_.

The use of growth-promoting chemicals accelerated the germination dynamics of only the freshly collected seeds (Figure 3). The lag phase from the beginning of incubation to the onset of germination was reduced to 2.1–3.3 days in seeds that ere imbedded with growth-promoting hormones and to 3.3 days in seeds that were imbedded with thiourea, while 50% germination was achieved about twice as fast as in the nontreated seeds. These results make growth-promoting hormones an attractive method to stimulate *A. artemisiifolia* germination without the need for stratification or a post-ripening period, as suggested earlier [16,17]. Seed stratification is a standard method of reducing physiological dormancy in *A. artemisiifolia*. However, we found that satisfactory germination can be achieved only if the seeds are exposed for at least 8 weeks to the stratification period, as reported in numerous previous studies [9,26,27,28,29,30]. Although the mechanism of stratification-induced release from dormancy is not known, germination may occur due to depletion of three silver-containing polypeptides during the stratification period [31] or possibly due to physical contact of the abrasive sand mixture with the seeds, weakening the embryo and thus stimulating germination [16]. However, in the present study, increasing the stratification period from 4 to 12 weeks did not promote the germination of freshly collected seeds that were maintained in a moist sand mixture (Figure 2).

The seedling stage of plants is the most vulnerable period in the life cycle of plants, as the growth of the seedlings directly affects the development and fitness of adult plants [32]. Therefore, here we investigated the emergence patterns and growth behavior of cold-stored and freshly collected *A. artemisiifolia* seeds that were imbedded or conditioned with growth-promoting hormones or thiourea. In most cases, the measured parameters (hypocotyl length, seedling weight, and height) showed negligible or only slightly significant differences between the cold-stored or freshly collected seeds. However, seeds that were conditioned in ethephon solution developed slightly longer hypocotyls or seedlings with lower heights and weights than the other treatments. Moreover, negligible deformations, i.e., development of internodes or deformation of first leaves, were observed in 3% of the plants that developed from ethephon or ethephon + GA_3_-treated seeds. Therefore, we conclude that the growth of *A. artemisiifolia* plants is not significantly affected when the seeds are treated with hormones or nitrogen fertilizers. However, since certain deformations were observed in the initial growth of *A. artemisiifolia* upon treatment with ethephon, the effect of these disturbances on further plant growth should be investigated in future studies, especially in resistance trials. Specifically, plants that are grown from ethephon-treated seeds should be treated with herbicides, and their response should be compared with plants that are emerging from nontreated seeds.

Our study clearly supports the theory that treatment with ethephon or ethephon + GA_3_ is the most effective method to overcome dormancy in *A. artemisiifolia* seeds when rapid experimental results are needed. Abnormal seedling growth was detected in *A. trifida* under the same conditions [16], but we did not observe such effects here. Prolonged cold storage should be avoided when the seed supply is limited because cold-stored seeds show lower viability than freshly collected ones. In the event that extensive cold storage is inevitable, *A. artemisiifolia* seeds can be conditioned in ethephon, GA_3_, or thiourea solution to promote germination. Nevertheless, the effects of these dormancy-breaking methods on plant growth should be further studied, especially after the use of herbicides.

## 4. Materials and Methods

### 4.1. Seed Collection and Storage

Mature seeds of *A. artemisiifolia* L. were collected in October 2018 and 2019 from plants that were grown in soybean fields in Požeška, Slavonia County, Badljevina (45°30′42″ N; 17°11′34″ E) and in Zagreb County, Topolje (45°42′36″ N; 16°20′24″ E). All of the seeds were collected from more than 20 grown *A. artemisiifolia* plants to adequately represent the genetic variability of a strictly allogamous species [33]. After each collection, the seeds were manually sorted by size and color, considering the relationship between size and germination [34] as well as their morphological and functional variability [35]. Only uniform seeds and those that were visually free of predator damage were used in the experiments. Seeds that were collected in 2018 were stored in a refrigerator at 4 °C for 12 months before treatment and are hereinafter referred to as “cold-stored” seeds. Seeds that were collected in 2019 were immediately used and are hereinafter referred to as “freshly collected” seeds. All of the seeds were subjected to continuous germination testing after 0, 2, 4, and 6 months of cold storage (i.e., 12, 14, 16, and 18 months of cold storage for the seeds that were designated as cold-stored seeds).

### 4.2. Laboratory Experiments

#### 4.2.1. Cold Stratification

The freshly collected and cold-stored seeds were first sterilized with 3% hydrogen peroxide for 6 min to inactivate pathogens, and then placed in glass Petri dishes (150 mm diameter, 25 mm height) containing 100 g of sterilized quartz sand and 50 mL distilled water. The Petri dishes were subsequently sealed with Parafilm to prevent evaporation and stored at 4 °C for 0 (non-stratified seeds), 2, 4, 6, 8, and 12 weeks. At each stratification time point, the *A. artemisiifolia* seeds were extracted from the sand and subjected to a 14-day germination test.

#### 4.2.2. Chemical Treatments

To assess the ability of chemical treatments to promote germination of freshly collected or cold-stored *A. artemisiifolia* seeds, nitrogen fertilizers (potassium nitrate (KNO_3_) and thiourea) and synthetic hormones (GA_3_ and ethephon) were applied as single treatments or in combination [16,17]. The seeds were either imbedded (24 h) or conditioned (48 h) in aqueous solutions of the growth promoters, or the aqueous solutions were added directly to the seed-containing Petri dishes (Table 3). For the treatment with nitrogen fertilizers, the seeds were imbedded in their aqueous solution for 24 h, or five of the aqueous solutions was added directly to the Petri dishes. In contrast, the hormone solutions were added directly to the Petri dishes.

Seed conditioning was performed based on a previous study on *A. trifida* seeds [16], where the seed embryo was released by incising the seed crown. However, embryo incision could not be applied to the *A. artemisiifolia* seeds because the embryo is fused with the seed coat. Therefore, the seed coat was manually removed with sandpaper (P 40) and the embryo was released during the conditioning process. The scarified seeds were placed in an Erlenmeyer flask that was filled with 200 mL of deionized water or a chemical solution. A flexible hose that was attached to an air supply (Flamingo, Crawfish 1800, Limburg, The Netherlands), which touched the bottom of the surface, was then placed in an Erlenmeyer flask. The opening at the top of the flask was sealed with Parafilm to prevent evaporation and to stabilize the flask tube. In addition, air was distributed evenly through the tube to prevent water or aqueous solution from leaking and to ensure the continuous movement of the seeds in the flask. After 48 h, the *A. artemisiifolia* seeds were removed from the flask, and a germination test was performed.

#### 4.2.3. Germination Tests

The germination tests were performed using a randomized complete block design with four replicates to determine the germination percentages of cold-stratified or chemically-treated seeds. The germination dynamics were assessed by counting the number of germinated seeds at 24 h intervals over a 14-day period [36].

The nontreated seeds were also tested after storage at 4 °C for 0, 2, 4, and 6 months (seed lot 2019) and 12, 14, 16, and 20 months (seed lot 2018) to evaluate germination over time without the application of dormancy-breaking methods.

For each germination test, 25 seeds were placed in sterile plastic Petri dishes (90 mm diameter) that were lined with filter paper (80 g m^−2^, 21/N, Munktell, Paul Marienfeld GmbH & Co. KG, Lauda-Königshofen, Germany) and 5 mL of distilled water or chemical solution was added. The Petri dishes were then sealed with Parafilm and placed in a climatic chamber (Memmert, UF 260, Schwabach, Germany) at a constant temperature of 25/15 °C with 70% humidity under standard photoperiod conditions (12 h:12 h) and a light intensity of 40–50 µmol m^−2^ s^−1^. The germinated seeds were counted daily for two weeks. Seed germination was defined as the development of 1 mm long radicles.

### 4.3. Greenhouse Experiment

Plant pots (12 cm diameter) were filled with a mixture of humus substrate (Potground H, Klasman, Geeste, Germany) and sterile soil in equal weight ratio (1:1). The soil, which was sampled from the Šašinovec Experimental Station at University of Zagreb (45°51′05.2″ N 68 16°10′34.1″ E), was classified as sandy clay with pH 7.74 (H_2_O), 7.04 (KCl), 4.22% humus, and 2.9% calcium carbonate and was sterilized before use at 100 °C for 30 min to eliminate all viable weed seeds [37].

*Ambrosia artemisiifolia* seeds were imbedded or conditioned with ethephon, GA_3_, ethephon + GA_3_, or thiourea solutions at the same concentrations as those that were used in the laboratory experiments (Table 3). Then, the seeds were sown at a depth of 1.5 cm [21,38] by placing the narrowest part from which the radicle develops towards the bottom of the pot. Afterward, the pots were placed in a greenhouse with an average ambient temperature of 15.8 °C and natural light conditions. An irrigation system (Water Control Master, GARDENA, Ulm, Germany) was used to maintain the humidity of the medium throughout the experiment. All of the plants were cultivated for 21 days and the number of emerged seedlings, as well as the hypocotyl length and the total plant height were measured every seven days. Growth deformities were also assessed qualitatively. The experiment was conducted using a complete block design in eight replicates with 20 seeds per pot.

### 4.4. Statistical Analysis

The laboratory and greenhouse experiment were performed twice with four and eight replicates each time, respectively. No differences were identified between the experiment using the t-test (*p* = 0.6638), and the data were combined for analysis. All the replicates were treated as a random effect, while the seed storage, stratification period, and seed treatments were treated as fixed effects. The normality and homogeneity of variance of all the data were evaluated, and an ANOVA was performed. The marginal means were estimated based on the best fitted linear model. The significance of differences between the estimated marginal means of variables was assessed using Tukey’s test. Differences with *p* < 0.05 were considered statistically significant. The statistical analysis was performed in R programming language and environment. The germination dynamics were quantified using a logistic function in the statistical program Bioassay 97, and the resulting germination time course was used to determine the time that was required for the germination of 10% (t_10_) or 50% (t_50_) of the sown seeds.

## Figures and Tables

**Table 1:** Analysis of variance for the effect of dormancy-breaking methods on *Ambrosia artemisiifolia* seeds.

Method			Df	SumSq	MeanSq	F-Value	Sig.
Seed storage		S	1	784	784.00	10.220	**
		T	3	6334	2111.33	27.522	***
		S × T	3	6650	2216.67	28.895	***
		Error	56	4296	76.71		
Stratification		S	1	14,292	14,291.5	88.6847	***
		T	5	55,441	11,088.3	68.8073	***
		S × T	5	1969	393.8	2.4435	*
		Error	83	13,375	161.1		
Chemical treatment	Direct addition	S	1	680	679.7	7.5818	**
		T	12	125,307	10,442.2	116.4800	***
		S × T	12	17,078	1423.2	15.8753	***
		Error	182	16,316	89.6		
	Conditioning	S	1	6062	6062.3	47.6656	***
		T	6	67,856	11,309.3	88.9209	***
		S × T	6	3613	602.6	4.7382	***
		Error	98	12,464	127.2		

S = Freshly collected and cold-stored seeds, T = treatment (type of germination stimulation: seed storage = months; stratification = weeks; chemical treatments = growth-promoting hormones or nitrogen fertilizer), Df = degrees of freedom, SumSq = sum of squares, MeanSq = mean of squares, Sig. = significant difference in means; (*) *p* = 0.05, (**) *p* = 0.01, (***) *p* < 0.001.

**Table 2:** Analysis of variance of hormones and nitrogen fertilizers treatments on the emergence, hypocotyl, and fresh biomass of *A. artemisiifolia* seedlings.

Variables		Df	SumSq	MeanSq	F-Value	Sig.
Emergence (%)	S	1	1464	1464.10	5.2093	*
	T	4	6808	1702.08	6.0561	***
	S × T	4	4359	1089.80	3.8776	**
	Error	150	42,158	281.05		
Hypocotyl length (cm)	S	1	1.3504	1.35038	8.7901	**
	T	4	1.0876	0.27190	1.7699	ns
	S × T	4	1.3802	0.34505	2.2460	ns
	Error	149	22.8902	0.15363		
Fresh biomass (g)	S	1	3.721	3.7210	5.7545	*
	T	4	13.298	3.3246	5.1414	***
	S × T	4	7.320	1.8301	2.8302	*
	Error	150	96.994	0.6466		

S = freshly collected and cold-stored seeds, T = treatment (growth-promoting hormones or nitrogen fertilizers imbedded or applied during conditioning), Df = degrees of freedom, SumSq = sum of squares, MeanSq = mean of squares, Sig. = significant difference in means; (ns) not significant, (*) *p* = 0.05, (**) *p* = 0.01, (***) *p* < 0.001.

**Table 3:** Treatments that were applied to freshly collected or cold-stored *Ambrosia artemisiifolia* seeds in the laboratory and the greenhouse.

Treatment	Concentration (mM)	Method		
		Imbibition (24 h)	Direct Addition	Conditioning (48 h)
**Distilled water ^d^**	-	**+**	**+**	+
**Ethephon ^a,d^**	1	**-**	**+**	+
**Gibberellic acid (GA_3_) ^b,d^**	1	**-**	**+**	+
**Thiourea ^c,d^**	263	**+**	**+**	+
**KNO_3_**	198	**+**	**+**	-
**Ethephon + GA_3_^d^**	1 + 1	**-**	**+**	+
**GA_3_ + thiourea**	1 + 263	**-**	**+**	-
**Ethephon + thiourea**	1 + 263	**-**	**+**	-
**Ethephon + KNO_3_**	1 + 198	**-**	**+**	-
**GA_3_ + KNO_3_**	1 + 198	**-**	**+**	-
**Thiourea + KNO_3_**	263 + 198	**-**	**+**	-
**Ethephon + GA_3_ + thiourea**	1 + 1 + 263	**-**	**+**	-
**GA_3_ + thiourea + ethephon + KNO_3_**	1 + 263 + 1 + 198	**-**	**+**	-

^a^ Ethrel (SL)—ethephon 480 g/L (Bayer). ^b^ Gibberellic acid for synthesis (Millipore). ^c^ Thiourea (CH_4_N_2_S), 99% Alfa Aesar ThermoFisher (Kandel) GmbH, Germany. ^d^ Treatments used in the greenhouse experiment.

## Data Availability

Not applicable.
